# Traditional Chinese medicine as a cancer treatment: Modern perspectives of ancient but advanced science

**DOI:** 10.1002/cam4.2108

**Published:** 2019-04-03

**Authors:** Yuening Xiang, Zimu Guo, Pengfei Zhu, Jia Chen, Yongye Huang

**Affiliations:** ^1^ College of Life and Health Sciences Northeastern University Shenyang China

**Keywords:** cancer stem cells, epigenetics, microenvironment, oncogene, traditional Chinese medicine

## Abstract

Traditional Chinese medicine (TCM) has been practiced for thousands of years and at the present time is widely accepted as an alternative treatment for cancer. In this review, we sought to summarize the molecular and cellular mechanisms underlying the chemopreventive and therapeutic activity of TCM, especially that of the Chinese herbal medicine‐derived phytochemicals curcumin, resveratrol, and berberine. Numerous genes have been reported to be involved when using TCM treatments and so we have selectively highlighted the role of a number of oncogene and tumor suppressor genes in TCM therapy. In addition, the impact of TCM treatment on DNA methylation, histone modification, and the regulation of noncoding RNAs is discussed. Furthermore, we have highlighted studies of TCM therapy that modulate the tumor microenvironment and eliminate cancer stem cells. The information compiled in this review will serve as a solid foundation to formulate hypotheses for future studies on TCM‐based cancer therapy.

## 1 INTRODUCTION

Cancer encompasses a group of diseases in which cells harbor the ability to exhibit uncontrolled proliferation with the potential to invade and undergo metastasis to other parts of the body. Cancer is a chronic health condition whose incidence and mortality rate are rapidly increasing in all regions of the world. In 2018, it is estimated that 18.1 million new cases of cancer and 9.6 million deaths occurred worldwide.^1^ Lung cancer, breast cancer, prostate cancer, and colorectal cancer are the cancers that have the highest incidence, whereas lung, colorectal, stomach, and liver cancers result in the greatest number of deaths due to cancer. The health burden of cancer is increasing in China, with approximately 3.6 million new cancer cases and 2.2 million deaths per year.^2^ In summary, cancer ranks as the leading cause of death in humans and is considered a serious threat to the improvement in life expectancy in every country of the world.

Recent decades have witnessed substantial improvements in the management of cancer, including both theoretical research and clinical practice. First of all, cancer is considered a genetic illness. As early as the 1990s, studies revealed the relationship between mutation of p53 and human cancer.^3^ During the past 30 years, the identity of a large number of genes giving rise to susceptibility to cancer has been established.^4^ Subsequently, epigenetics has added a great deal of complexity to our understanding of the regulation of the cancer genome. Increasing evidence has confirmed that genetic and epigenetic changes contribute to the initiation of cancer and its development and subsequent progression. The cancer stem cell concept was first proposed in the 1800s, but the first isolation of cancer stem cells was reported in 1994.^5^, ^6^ Cancer stem cells, with the capability for self‐renewal and potential for proliferation, are possibly responsible for tumor initiation, progression, migration, invasion, and distant metastases.^7^, ^8^ Cancer stem cells account for the resistance to chemotherapeutic agents during tumor therapy, and/or contribute to cancer recurrence. In addition to cancer stem cells, the “tumor microenvironment” is an important additional concept for cancer initiation and development. Tumors have been recognized as organs with complex interactions and there are a number of cell types that contribute to the biology of tumors.^9^ The cross‐talk between tumor cells and their microenvironment is an important target for cancer therapy. In general, the failure of chemotherapy to cure patients with a malignancy is due to the presence of intratumoral heterogeneity and molecular complexity. Genetic mutations, the presence of cancer stem cells, and interactions with the microenvironment are considered the major factors accounting for intratumoral heterogeneity and acquired chemotherapeutic resistance.^8^

During recent decades, several forms of complementary and alternative medicine have been utilized to defeat cancer worldwide. Traditional Chinese medicine (TCM) has been widely accepted as a mainstream form of complementary and alternative therapy with beneficial effects for cancer patients in China.^10^, ^11^ In fact, TCM has been commonly used in Asia for thousands of years. The largest application category of TCM is Chinese herbal medicine (CHM) which comprises sliced herbal and Chinese patented drugs.^12^ A large number of cancer patients have used CHM as an alternative therapy because of its effectiveness and lack of serious side effects. For example, in nonsmall‐cell lung cancer (NSCLC) therapy, CHM has been shown to have fewer toxic effects, provide enhanced quality of life, prolong survival rate, and improve immediate tumor response and Karnofsky performance score.^13^, ^14^, ^15^ Additionally, several representative CHM‐derived phytochemicals, namely curcumin, resveratrol, berberine, dioscin, baicalein, wogonin, silibinin, quercetin, tanshinone IIA, and celastrol have been studied in detail, their administration demonstrated to exhibit antitumor effects in many cancers.^16^, ^17^, ^18^

**Figure 1:**
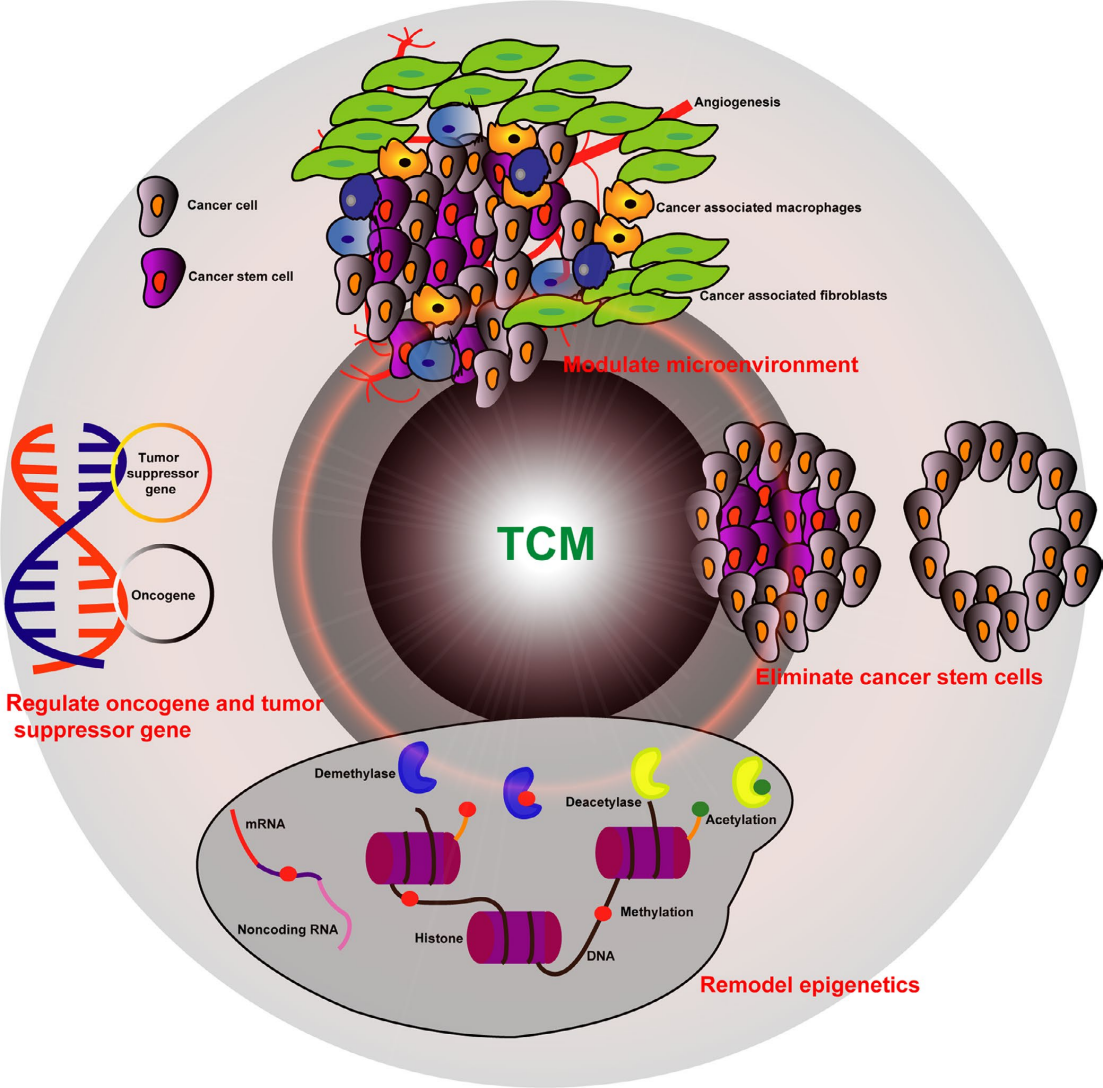
Schematic diagram demonstrating the application of TCM in cancer therapy based on genetics, epigenetics, tumor microenvironment, and cancer stem cells

According to the theory of Chinese medicine, the occurrence of illness is due to the disturbance of two opposing forces of energy, Yin and Yang. To alleviate symptoms of disease, Chinese medicine aims to restore the harmony of Yin and Yang. Thus, people often have the impression that TCM therapy is mysterious. However, several reviews have described the application of TCM therapy on cancer treatment,^17^, ^19^, ^20^, ^21^, ^22^ and therefore this review attempts to comprehensively illustrate the underlying mechanisms of TCM‐based CHM on cancer therapy from the point of view of applicable genetic and epigenetic theory, cancer stem cells, and the tumor microenvironment (Table 1; Figure 1).

**Table 1:** Selected observations from studies of TCM involving genes, epigenetics, tumor microenvironment and cancer stem cells

Category	Genes (oncogene and tumor suppressor gene)	Epigenetics (DNA and histone modification)	Microenvironment	Cancer stem cells
Curcumin	P53↑^23^, ^24^; PTEN↑^25^; c‐Myc↓^26^; k‐Ras↓^27^; Bcl‐2↓^28^; EGFR↓^27^	DNMT1↓^29^; DNMT1, 3A and 3B↓^30^; pan‐HDAC inhibitor 31	Angiogenesis↓^32^; Cancer‐associated fibroblasts↓^33^, ^34^; Cytotoxic effect of NK cells↑^36^; CD8(+) T cells↑, Tregs↓^37^	Colon cancer stem cells↓^38^; Acute myeloid leukemia cancer stem cells↓;^39^ Glioblastoma stem cells↓^40^; Liver cancer stem cells↓^41^; Breast cancer stem cells↓^42^, ^43^; Head and neck squamous cell carcinoma cancer stem cells↓^44^; Prostate cancer stem cells↓^45^
Resveratrol	p53 phosphorylation↑^46^; mutant p53↓^47^; PTEN↑^25^; c‐Myc↓^48^; Bcl‐2↓^49^; k‐Ras↓^50^; EGFR phosphorylation↓^51^	DNMT1, DNMT3A, DNMT3B↓^52^; HDAC1, HDAC2↓ 53	CD8(+) T cells↑^54^; Angiogenesis↓^54^; Cancer‐associated fibroblasts↓^48^, ^55^, ^56^	Glioma stem cell↓^57^; Cervical cancer stem cells↓^58^; Breast cancer stem cells↓^59^; Colon cancer stem cells↓^60^; Leukemia stem cell↓^61^
Berberine	P53↑^62^; PTEN↑^63^; c‐Myc↓^64^; Bcl‐2↓^65^; EGFR↓^66^	DNMT1, DNMT3B↓^67^; CREBBP, EP300, SIRT3, KDM6A, SETD7↑^68^; HDAC8, H3K4me3, H3K27me3, H3K36me3↓^68^; pan‐HDAC inhibitor 65	Angiogenesis↓^69^; Cancer‐associated fibroblasts↓^70^	Oral squamous cell carcinomas‐cancer stem cells↓^71^; Leukemic stem cells↓^72^
Dioscin	p53↑^73^; Bcl‐2↓^73^, ^74^; VEGF‐A↓^74^	TET1↓, TET2↑, TET3↑, DNMT3A↑ 76	Macrophage sensitivity↑^77^	Osteosarcoma stem cells↓^75^; Prostate cancer stem cells↓^74^
Baicalein	Bcl‐2↓^78^; p53↑, VEGF‐A↓, EGFR↓^79^; PTEN↑^80^; c‐Myc↓^81^		Tumor‐associated macrophages polarization↓^82^; Inflammatory microenvironment↓^78^, ^83^, ^84^	Liver cancer stem cells↓^85^; Pancreatic cancer stem cells↓^86^
Wogonin	p53↑, VEGF‐A↓, Bcl‐2↓^87^; c‐Myc↓^88^	HDAC1, HDAC2↓^88^	Inflammatory microenvironment↓^78^, ^89^	Osteosarcoma cancer stem cells↓^90^
Silibinin	p53 acetylation↑^91^; PTEN↑^92^, ^93^; c‐Myc↓^94^; Bcl‐2↓^93^, ^95^; VEGF, EGFR↓^93^	DNMT1↓^96^; H3K27me3↑^97^; SIRT1↓^91^; acH3, acH4↑^92^, ^98^; HDAC1‐3↓^98^	Cancer‐associated fibroblasts↓^99^, ^100^; Tumor‐associated myeloid‐derived suppressor cells↓^101^	Hepatocellular carcinoma stem cells↓^95^; Glioblastoma stem cells↓^93^; Head and neck squamous cell carcinomas stem cells↓^102^; Breast cancer stem cells↓^103^; Colon cancer stem cell↓^104^; Lung cancer stem cells↓^105^
Quercetin	p53, p53 phosphorylation↑^106^; PTEN↑^107^; c‐Myc↓^108^; k‐Ras↓^109^; Bcl‐2↓^106^, ^110^, ^111^; VEGF, EGFR↓^112^	DNMT1↓^113^, ^114^; DNMT3a↓^114^; HDAC1↓^113^; acH3↑, acH4↑, HAT activity↑, HDAC activity↓^115^	Acidity of tumor microenvironment↓^112^; Cancer‐associated fibroblasts↓^116^; Inflammatory microenvironment↓^117^	Prostate cancer stem cell↓^118^, ^119^; Breast cancer stem cells↓^110^, ^120^, ^121^; Glioblastoma stem cells↓^122^; Gastric cancer stem cells↓^111^; Colorectal cancer stem cells↓^123^; Pancreatic cancer stem↓^124^
Tanshinone IIA	p53↑^125^; PTEN↓^126^; c‐Myc↓^127^; Bcl‐2↓^125^, ^127^; VEGF↓^127^, ^128^; EGFR↓^126^		Angiogenesis↓^128^; Inflammatory microenvironment↓^129^	Breast cancer stem cells↓^130^, ^131^; HepG2 cancer stem cells↓^132^; Glioma stem cells↓^133^
Celastrol	p53 phosphorylation↑^134^; c‐Myc↓, Bcl‐2↑^135^; VEGF↑^136^; VEGF↓^137^; EGFR↓^138^		Remodeling fibrotic and immunosuppressive tumor microenvironment;^139^ Inflammatory microenvironment↓^140^	Colon cancer stem cells↓^141^; Leukemia stem cells↓^142^
Pterostilbene	p53↑^143^; PTEN↑^144^; c‐Myc↓^145^; VEGF↓^146^	demethylation of MAML2 enhancer 147; acH3↑^146^; HDAC1↓^144^; HDAC5 phosphorylation↑^148^	Tumor‐associated macrophages↓^149^, ^150^	Breast cancer stem cells↓^145^, ^149^; Glioma stem cells↓^151^; Hepatoma stem cells↓^152^

Note

↑Indicates increased or activated; ↓indicates decreased or inactivated.

## 2 TCM IN CANCER THERAPY

The principal strategies of cancer treatment include surgical resection, radiotherapy, and chemotherapy. Other means of cancer therapy, such as immunotherapy and targeted therapy, have led to significant breakthroughs in curing cancer in recent years. Over recent decades, a number of clinical and laboratory studies have been conducted with the aim of establishing the effectiveness of TCM in treating cancer. Several CHM‐derived compounds have exhibited anticancer properties that inhibit the development, proliferation, angiogenesis, and metastasis of human cancer. In particular, resveratrol, curcumin, and berberine have all been evaluated in a number of clinical trials for the treatment of many types of cancer.^153^ In most cases, TCM is taken as an adjuvant therapy for cancer.

Surgical resection remains the first choice for treatment of nonmetastatic cancer. In a recent study of 345 patients who had undergone surgical resection for locally advanced colon adenocarcinoma, the patients who received the CHM catalpol as a treatment had superior outcomes in many respects regarding efficacy, safety, and cost of therapy.^154^ It suggests that TCM is an alternative method that improves the survival and quality of life of cancer patients.

There have been many reports published that show patient outcomes after combining TCM with chemotherapy. PHY906, formulated using the herbs *Scutellaria baicalensis*, *Ziziphus jujuba*, *Paeonia lactiflora,* and *Glycyrrhiza glabra,* has been reported to reduce the adverse effects of capecitabine in advanced hepatocellular carcinoma patients in a phase I/II clinical study.^22^, ^155^ It was shown that oral CHM improved the outcomes of chemotherapy when considering quality of life, anemia, and neutropenia in patients with NSCLC.^13^ Curcumin, a polyphenol and bioactive metabolite extracted from the rhizomes of *Curcuma longa* L., is widely used in TCM.^156^ A study of pancreatic cancer demonstrated that combination therapy of daily 8 g oral curcumin with gemcitabine‐based chemotherapy was both safe and feasible for use in patients.^157^ Cisplatin is considered to be among the most effective chemotherapeutic drugs. In human hepatoma cells, resveratrol was shown to improve cisplatin toxicity via an apoptosis‐dependent mechanism.^158^ The principal aim of chemotherapy was to induce apoptosis in cancer cells, inevitably resulting in various adverse effects, such as gene mutation, cellular toxicity, and drug resistance. TCM can possibly perform an important role by reducing the adverse effects brought about by chemotherapy, thus improving therapeutic outcome and quality of life for patients.

Radiotherapy is an additional important therapeutic technique in the fight against cancer. It is reported that approximately half of women with breast cancer receive radiotherapy in the most developed countries.^159^ TCM operates as a radiosensitizing agent for cancer treatment. For example, curcumin can sensitize nasopharyngeal carcinoma cells to radiation through the regulation of ROS generation, Jab1/CSN5, and noncoding RNA expression.^160^ Resveratrol and berberine have also been shown to enhance radiosensitivity in nasopharyngeal carcinoma cells.^161^, ^162^ Furthermore, radiotherapy has the problem that it inevitably involves the exposure of noncancerous tissues to radiation, leading to side effects such as xerostomia, hepatotoxicity, or pneumonitis. TCM is a promising complementary therapy, having been used inconsistently in the management of radiotherapy‐induced adverse effects. The results of a systematic review indicated that treatment using TCM reduced the incidence of radiation xerostomia which is observed in 80% of head and neck cancer patients receiving radiotherapy.^163^ TCM has been suggested in an earlier report to be an effective adjunctive therapy for the reduction in the incidence of chronic hepatitis in breast cancer patients receiving radiotherapy and/or chemotherapy.^164^ Curcumin has been shown to significantly prolong the median survival time of mice bearing esophageal squamous cell carcinoma while exposed to radiation therapy.^165^ In summary, treatment using TCM can be effective in enhancing radiosensitization and reducing side effects.

Cancer immunotherapy was ranked as 2013’s Breakthrough of the Year by *Science*.^166^ Recently, regulation of the immune system to eliminate cancer cells has succeeded in clinics through the use of immune checkpoint therapy that utilizes blocking antibodies to cytotoxic T lymphocyte antigen‐4 (CTLA‐4) and programmed death‐1 (PD‐1), and via chimeric antigen receptor (CAR) T cells.^167^ TCM can also offer a potential immunomodulatory regimen for the treatment of a number of diseases, including cancer. In patients with colon cancer, the frequency of T‐helper 1 cells can be greatly enhanced after curcumin therapy.^168^ Combining curcumin‐polyethylene glycol conjugate with a vaccine was shown to significantly promote cytotoxic T‐lymphocyte response and interferon‐γ release in vivo.^169^ Resveratrol is among the most‐studied natural phytochemicals worldwide because its potential therapeutic effects are relevant to the treatment of many diseases, including cancer. In a mouse renal tumor model, low dose resveratrol administration was shown to inhibit tumor growth by modulation of CD8(+) T cells.^54^ Therefore, many TCM compounds may be promising candidates for use in combination with immunotherapy as a treatment for cancer.

## 3 GENES PARTICIPATE IN TREATMENTS USING TCM

Genetic aberrance is a common phenomenon in cancer. For example, there are commonly recognized gene mutations in KRAS, TP53, and EGFR, and rearrangements in ALK and ROS1 in lung cancers.^170^ Mutations in oncogenes and tumor suppressor genes invariably constitutively activate downstream signaling pathways, sustaining carcinogenesis and cancer progression together.

### 3.1 Impact on tumor suppressor genes

TP53 is a well‐known tumor suppressor gene and mutant p53 protein is frequently expressed in abundance in numerous cancers. p53 can induce cell cycle arrest, apoptosis, or senescence, depending on the specific context, including DNA damage, hypoxia, and oncogene activation, etc*.* 171 Several studies have demonstrated that curcumin regulates the p53 signaling pathway. In hepatic stellate cells, curcumin induces senescence via modulation of p53 expression.^23^ In gastric cancer, curcumin treatment enhances the expression of p53 and p21.^24^ Resveratrol is also reported to induce p53 phosphorylation at serine 20 that activates p53‐target genes such as PUMA and BAX, thus restoring apoptosis in MCF7 cells resistant to cisplatin.^46^ Furthermore, intracellular aggregation of mutant p53 leads to its inactivation. Resveratrol has been shown to reduce mutant, but not wild‐type, p53 protein aggregation.^47^ Berberine induces G2/M phase cell cycle arrest via promotion of p53 and p21 expression, and suppression of retinoblastoma (Rb), cyclin B1, cyclin‐dependent kinase 1, and cdc25c expression in HTB‐94 chondrosarcoma cells.^62^ In addition to p53, Rb, PTEN, and APC are also important tumor suppressor genes. Curcumin treatment restores the expression of the tumor suppressor proteins p53, pRb, and PTEN13 in cervical cancer cells.^172^ Thus, evidence suggests that there are TCMs that reactivate the expression of tumor suppressor genes that fight cancer.

### 3.2 Regulation of oncogene expression

An oncogene, the mutation and/or overexpression of which is often observed in cancer, has the potential to cause cancer. Chromosomal rearrangements, mutations, and gene amplification can activate oncogenes, conferring a growth advantage or increased survival to these cells. Studies of the changes in Myc, Ras, and Bcl2 oncogenes provided the first wave of evidence that cancer arises from somatic genetic aberrance.^173^ Oncogenes can be classified into several categories, comprising transcription factors (eg, Myc), growth factors (eg, PDGF), growth factor receptors (eg, VEGFR, EGFR), signal transducers (eg, PI3K, Akt, mTOR), cytoplasmic tyrosine kinases (eg, Src family), cytoplasmic serine/threonine kinases (eg, Raf kinase), or regulators of apoptosis (eg, Bcl2).

Myc is a well‐recognized oncogene, and studies have revealed that Myc expression in both BxPC3 and gemcitabine‐resistant BxPC3 cells can be suppressed by treatment with curcumin.^26^ Resveratrol has been shown to significantly decrease the phosphorylation of Her‐2 and EGFR and the expression of Erk in ovarian cancer cell lines.^51^ A separate study demonstrated that resveratrol was able to enhance the expression of Caspase 3 and 9 and decrease the expression of Bcl2, Ras, Raf, MEK, and ERK1/2 in a dose‐dependent manner in human colon cancer.^49^ In colon tumor cells, berberine treatment was demonstrated to suppress EGFR expression via enhancement of Cbl activity.^66^ As for the regulation of apoptosis regulators, curcumin, resveratrol, and berberine have all been shown to trigger apoptosis and increase the ratio of Bax/Bcl‐2 expression.^28^, ^49^, ^65^ In fact, apoptosis has wide involvement in the antitumor effects of treating with CHM.

## 4 EPIGENETIC BASIS OF TCM

Epigenetics is defined as the study of heritable alterations in gene expression that retain the original DNA sequence.^174^ Epigenetic modifications play a key role in the regulation of the expression of genes related to cell development and differentiation. Epigenetic abnormalities are shown to closely associate with the process of cancer initiation and progression.^175^ In various cancer models, aberrant DNA and histone modifications have been shown to silence tumor suppressors or promote oncogenes.^176^ The three major epigenetic modifications, namely DNA methylation, changes to chromatin, and noncoding RNA profiles, are discussed in this chapter.

### 4.1 DNA methylation and demethylation

The most well‐studied epigenetic marker is DNA methylation, in which a methyl group is added to the fifth position of the six‐membered ring of cytosine which forms 5‐methylcytosine (5‐mC). DNA methylation is mediated by a number of enzymes, including cytosine‐5 methyltransferase and DNA methyltransferases (DNMT1, DNMT2, DNMT3a, DNMT3b, and DNMT3L). DNA methylation participates in numerous biological events, including embryonic development, gene expression, genomic imprinting, and disease pathogenesis^177^ Tumorigenesis is thought to result from the most extreme aberrant DNA methylation events, of either tumor suppressor gene hypermethylation or oncogene hypomethylation.

Several CHM‐derived phytochemicals have been described as regulators of cellular epigenetic events, including DNMT activity, in a variety of cancers. In breast cancer, curcumin can decrease DNA methylation and DNMT1 expression, but in particular it can decrease methylation of the promoter of ras‐association domain family protein 1A that reactivates its expression.^29^ It appears that resveratrol exerts a considerably greater effect on DNA methylation, with treatment of a number of types of cancer cells causing a reduction of DNMT enzymatic activity and expression of DNMT1, DNMT3A, and DNMT3B in a number of cancer cells.^52^ Three hundred and thirty‐eight cancer‐related genes were shown to be hypermethylated and 92 cancer‐related genes hypomethylated in MDA‐MB‐231 cells exposed to resveratrol for 24 h.^178^ Berberine has also been shown to change the pattern of DNA methylation. In human multiple melanoma cell line U266, berberine treatment inhibited the expression of DNA methyltransferases DNMT1 and DNMT3B, thus triggering hypomethylation of p53 and modifying the p53‐dependent signaling pathway.^67^

5‐mC can be deactivated by a family of α‐ketoglutarate‐dependent dioxygenases, the ten‐eleven translocation (TET) proteins (TET1, 2, and 3). The TET proteins are responsible for the sequential oxidation of 5‐mC into 5‐hydroxymethylcytosine (5‐hmC), then 5‐formylcytosine (5‐fC), and finally 5‐carboxylcytosine (5‐caC).^179^ 5‐fC and 5‐caC are short‐lived intermediates of an active DNA demethylation pathway, while 5‐hmC can possibly be regarded as an independent epigenetic marker. The global loss of 5‐hmC has recently been proposed as a novel candidate biomarker of the presence of an aggressive tumor with both diagnostic and prognostic implications. Reduced 5‐hmC levels are observed in many cancers, including prostate,^179^ ovarian,^180^ melanoma,^181^ and lung 182 cancer. In animal disease models, the growth of aggressive tumors can be thwarted by restoring 5‐hmC levels via IDH2 or TET2 overexpression.^181^ Therefore, modulation of the DNA demethylation process could also be an important target for cancer treatment.

As far as can be ascertained, investigations of the relationship between TCM and DNA demethylation are limited. Dioscin, a natural steroid saponin, has been identified in CHM. Dioscin is reported to significantly suppress the proliferation of colon cancer cells via regulation of reactive oxygen species generation, and modulation of the p38 MAPK and JNK signaling pathways.^183^ In breast cancer, dioscin has also been shown to suppress cell proliferation, invasion, and migration.^76^ In addition, the expression of TET2 and TET3 is upregulated, and TET1 downregulated in cells treated with dioscin.^76^ Resveratrol has been shown to enhance the enzymatic activities of IDH2.^184^ It appears that a number of TCM phytochemicals are involved in the regulation of 5‐hmC formation and deactivation. In fact, vitamin C, in high abundance in fruit and vegetable, has been shown to have wide participation in the process of DNA hydroxymethylation and so exerts antitumor function.^185^, ^186^ Therefore, it is logical to speculate that an important function of TCM in cancer treatment is the regulation of TET‐dependent DNA demethylation.

### 4.2 Histone modification

Histone modification is the second most important category of epigenetic modification. Any chemical reaction, including methylation, acetylation, phosphorylation, ubiquitylation, or sumoylation, can change the overall structure of chromatin, thus affecting gene expression by modulation of the condensation of DNA or recruitment of effector molecules that regulate the expression of downstream genes.^187^, ^188^ Aberrant histone modification can change secondary DNA structure leading to abnormal expression of cancer‐related genes. Therefore, in many cancer studies, attempts have been made to restore the histones to their original state prior to modification. Inhibition of DNA and histone methylation by decitabine (a potent inhibitor of DNA methylation) and 3‐Deazaneplanocin‐A (an inhibitor of histone methyltransferase EZH2) causes a synergistic antineoplastic action and activation of many tumor suppressor genes in myeloid leukemic cells.^189^ Treatment with curcumin has been shown to attenuate the expression of PRC2 subunit EZH2 in many cancer cells.^26^, ^190^ Resveratrol can inhibit the activity of lysine‐specific demethylase‐1, an enzyme that regulates histone methylation, and so prevent histone modulation.^191^ Berberine treatment leads to an increase in histone acetyltransferases CREBBP and EP300, histone deacetylase SIRT3, histone demethylase KDM6A, histone methyltransferase SETD7, and a decrease in histone acetyltransferase HDAC8, and histone methyltransferases WHSC1I, WHSC1II, and SMYD3 in multiple myeloma cells.^68^ In addition, treatment with berberine results in the downregulation of H3K4me3, H3K27me3, and H3K36me3 expression.^68^ However, as summarized in Table 1, reports demonstrating the ability of treatment using TCM to influence histone methylation are limited.

Notably, among posttranslational covalent modifications of histones, histone acetylation is most extensively studied. Histone acetylation is deeply involved in transcriptional activation and is regulated by two opposing classes of enzymes, histone acetyltransferases (HATs) and deacetylases (HDACs).^192^ HATs perform acetylation of specific lysine residues and induce enhanced gene transcription. HDACs enable the negatively charged DNA to bind to nucleosome proteins by removing acetyl groups from the positively charged histone lysine residues, thus performing epigenetic regulation of gene expression. The balance between acetylation and deacetylation through the enzymatic activity of HDACs is often dysregulated in numerous diseases, including cancer. Therefore, HDACs represent a promising target for anticancer therapy and HDAC inhibitors are widely used clinically as anticancer agents to alter the regulation of histone and nonhistone proteins. Curcumin is considered a natural pan‐HDAC inhibitor as it can downregulate HDAC activity and suppress the expression of HDAC types 1, 2, 3, 4, 5, 6, 8, and 11 in numerous cancer cell lines.^31^ Resveratrol can also modulate the activity of HDACs both in vitro and in vivo. Resveratrol upregulates the acetylation and reactivation of tumor suppressor gene PTEN via suppression of the MTA1/HDAC complex in prostate cancer.^53^ p53 acetylation and so activation occurs as a result of resveratrol's role in the inhibition of MTA1 that leads to increased p53 acetylation.^193^ Additionally, berberine has been shown to repress total HDAC but specifically class I, II, and IV HDAC activity via hyperacetylation of histones, downregulation of oncogene expression (TNF‐α, COX‐2, MMP‐2, and MMP‐9), and upregulation of the expression of tumor suppressor genes (p21 and p53).^65^ It appears that histone acetylation and deacetylation have broad involvement in cancer treatment using TCM.

### 4.3 Noncoding RNA regulation

In mammalian cells, the majority of RNAs do not have protein‐coding function. Transcription eventually produces many small mature RNAs, including microRNAs, piRNAs, snoRNAs, and tiRNAs, but some transcripts are processed into long noncoding RNAs (lncRNAs).^194^

Gene expression can be regulated at the posttranscriptional level by varieties of microRNA which normally comprise 20‐22 nucleotides. MicroRNAs generally block the translation of mRNA into protein through their binding to the 3′‐untranslated region of target mRNAs via imperfect complementary binding. Dysregulation of microRNAs has been observed throughout various stages of cancer.^195^ They can regulate many signaling pathways involved in cancer initiation, development and progression, therefore, microRNAs can be used as an important diagnostic and prognostic biomarker. A large number of microRNAs have been shown to have involvement in cancer therapy using TCM. The Chinese herbal formulation JP‐1 activates p53 and downstream p21 and BAX proteins in addition to miR‐34a in A549 lung adenocarcinoma cells, leading to inhibition of proliferation, induction of apoptosis, a reduction in colony formation, and repression of migration.^196^ It has been suggested that miR‐34a is a master p53 downstream tumor suppressor in cancer, participating in cell cycle arrest, resistance to apoptosis, and progression of epithelial‐mesenchymal transition (EMT).^197^, ^198^ Curcumin induces apoptosis in multiple myeloma via modulation of the EZH2‐miR‐101 regulatory feedback loop.^190^ Resveratrol enhances the expression of major histocompatibility complex class I chain‐related proteins A and B via inhibition of the c‐Myc/miR‐17 pathway in breast cancer cells.^199^ Berberine also regulates the expression of microRNAs in various cancers. In SGC‐7901 cells treated with berberine, 347 microRNAs were found to be upregulated and 93 downregulated following miRNA sequencing.^200^ Regulation of microRNA expression is possibly a mechanism underlying many TCM therapies.

lncRNAs, which are defined as having a length of more than 200 nucleotides and lacking protein‐coding capability, are involved in the regulation of cell proliferation, differentiation, and chromosome modification.^201^ There is mounting evidence confirming that lncRNAs constitute an important component of tumor biology (Table 2). It has been found that curcumin sensitizes chemoresistant cancer cells by suppressing the expression of the lncRNA PVT1.^26^ Curcumin inhibits the expression of lncRNA H19 in a concentration‐dependent manner, and its ectopic expression reverses curcumin‐induced cell apoptosis and decreases p53 expression.^28^ The lncRNA MALAT1 serves as a potential marker of survival in stage 1 NSCLC patients and is downregulated following resveratrol treatment.^202^ It has been reported that the expression of a total of 538 lncRNAs can be reversed by treatment with berberine,^203^ indicating that the global effect of berberine is to modulate lncRNA expression. However, published literature that documents the involvement of lncRNAs in the antitumor effects of berberine is limited. As far as can be ascertained, studies of the underlying mechanisms involved in the expression of lncRNAs for many TCM therapies are sparse.

**Table 2:** Cross‐talk between lncRNA expression and treatment with curcumin or resveratrol

TCM agent	lncRNA	Cancer	Major findings (reference)
Curcumin	UCA1	Lung cancer	Curcumin inhibits the expression of lncRNA UCA1; lncRNA UCA1 overexpression abolishes the effect of curcumin on cell apoptosis^204^
Curcumin	GAS5	Breast cancer	Combination of lncRNA GAS5 down‐regulation and dendrosomal curcumin treatment shows lower percentages of apoptotic cells and a higher level of penetration through membranes compared with dendrosomal curcumin treatment alone^205^
Curcumin	PINT	Acute lymphoblastic leukemia	Curcumin induces the expression of lncRNA PINT^206^
Curcumin	PVT1	Pancreatic cancer	PVT1 is the only lncRNA significantly down‐regulated by curcumin; curcumin sensitizes chemoresistant cancer cells by inhibiting the expression of the PRC2 subunit EZH2 and its related lncRNA PVT1^26^
Curcumin	ROR	Prostate cancer	Curcumin induces high miR‐145 expression and inhibits the expression of lncRNA ROR^207^
Curcumin	PANDAR	Colorectal cancer	Curcumin increases lncRNA PANDAR expression; silencing lncRNA PANDAR in curcumin‐treated cells induces apoptosis and greatly attenuates senescence possibly by stimulating the expression of PUMA^208^
Curcumin	MEG3	ovarian cancer	Curcumin induces demethylation in the promoter region of lncRNA MEG3; lncRNA MEG3 restoration by curcumin significantly reduces miR‐214^209^
Curcumin	MEG3, HOTAIR	Hepatocellular cancer	Curcumin induces lncRNA MEG3 overexpression and lncRNA HOTAIR downregulation^30^
Curcumin	HOTAIR	Renal cell carcinoma	Curcumin inhibits the migration of cells with high lncRNA HOTAIR expression^210^
Curcumin		Nasopharyngeal carcinoma	Curcumin significantly reverses irradiation‐induced lncRNAs (AF086415, AK095147, RP1‐179N16.3, MUDENG, AK056098 and AK294004)^211^
Curcumin	H19	Gastric cancer	Curcumin suppresses lncRNA H19 expression in a concentration‐dependent manner; ectopic expression of lncRNA H19 reverses curcumin‐induced proliferative inhibition and apoptosis, and downregulated p53 expression; curcumin decreases the expression of the c‐Myc oncogene and exogenous c‐Myc protein reverses curcumin‐induced downregulation of lncRNA H19 expression^28^
Resveratrol	NEAT1	Multiple myeloma	Resveratrol represses lncRNA NEAT1 expression; resveratrol counteracts positive effects of lncRNA NEAT1 overexpression on multiple myeloma cell migration and invasion through the Wnt/β‐catenin signaling pathway^212^
Resveratrol	AK001796	Lung cancer	lncRNA AK001796 is downregulated in resveratrol‐treated lung cancer cells^213^
Resveratrol	MALAT1	Colorectal cancer	Resveratrol down‐regulates lncRNA MALAT1, resulting in decreased nuclear localization of β‐catenin thus attenuated Wnt/β‐catenin signaling, which leads to the inhibition of colorectal cancer cell invasion and metastasis^202^

## 5 TCM MODULATES THE TUMOR MICROENVIRONMENT

Tumor microenvironment is described as the cellular and physical environment surrounding primary tumors, comprising fibroblasts, endothelial, immune, and inflammatory cells, components of the ECM and soluble factors.^214^ The tumor microenvironment allows cross‐talk between the cancer and host cells, promotes functions such as angiogenesis, tumor‐promoting inflammation, and immune escape. Modulation of the activity of the immune system, especially the immune component of the tumor microenvironment, promises considerable potential for the treatment of cancer. Over the past decade, it has been shown that multiple factors within the tumor microenvironment, including immune cell infiltration prior to therapy, can influence the outcome of immunotherapy.^215^ As discussed earlier, a large number of TCM agents exhibit promising therapeutic effects relating to immunotherapy, indicating that these TCMs possibly contribute to the modulation of the tumor microenvironment. In a mouse renal tumor model, resveratrol was shown to enhance the accumulation of activated CD8(+) T cells in the tumor microenvironment and increase the expression of Fas ligand so as to exert cytotoxicity.^54^ The regulatory subpopulation of cells (CD4(+)CD25(+)FoxP3(+)Treg cells) is among the most potent and well‐studied tumor‐induced immunosuppressive phenotype that exists within the tumor microenvironment. The resveratrol analogue HS‐1793 has been shown to modulate this subpopulation.^216^ Toll‐like receptors perform key roles in the host immune system and participate in the development of cancer. Curcumin, resveratrol, and berberine can suppress activation of the toll‐like receptor 4 signaling pathway which is closely related to the inflammatory response and cancer progression.^217^ Angiogenesis is a fundamental step in tumor proliferation and expansion. Curcumin in combination with (−)‐epigallocatechin‐3‐gallate markedly reduces tumor growth and microenvironment‐induced angiogenesis via modulation of the JAK/STAT3/IL‐8 pathway in colorectal carcinoma.^32^ Treatment with resveratrol can downregulate angiogenesis via decreased expression of VEGF in the tumor microenvironment.^54^ Berberine has been shown to reduce tumor‐induced angiogenesis both in vitro and in vivo.^69^ Cancer‐associated fibroblasts, a major component of the tumor microenvironment, stimulate tumor progression through cross‐talk. Thus, inhibition of cancer‐associated fibroblasts by curcumin reduces the migration and metastasis of pancreatic cancer cells.^33^ Resveratrol treatment suppresses proliferation, migration, and invasion through downregulation of cyclin D1, c‐Myc, MMP‐2, and MMP‐9 expression in human breast cancer cells treated with cancer‐associated fibroblast‐conditioned media.^48^ Intervention with berberine is effective in inhibiting cell proliferation and tumorigenicity via suppression of STAT3 activation induced by cancer‐associated fibroblasts in nasopharyngeal carcinoma cells.^70^ Composition of the intestinal microbiota is closely associated with the development of colorectal cancer via their interaction with the surrounding environment. Berberine is reported to rescue *Fusobacterium nucleatum*‐induced colorectal tumorigenesis by modulation of tumor microenvironment and interference in the activation of tumorigenesis‐related pathways.^218^ As shown in Table 1, there is evidence that all TCM compounds summarized differentially exert positive effects against the tumor microenvironment. However, it is clear that more effort is required to ensure effective and systematic use of TCM in controlling the tumor microenvironment.

## 6 TCM AS A TREATMENT FOR CANCER STEM CELLS AND EMT CELLS

### 6.1 TCM against cancer stem cells

Despite the survival of cancer patients improving significantly, a large proportion continues to suffer recurrence. The failure of many therapeutic agents used to treat cancer patients is possibly due to primary or acquired resistance to chemotherapeutic or biologic agents. Intratumoral heterogeneity of many cancers, considered to be the principle reasons for therapeutic resistance include genetic mutation, interactions with the microenvironment, and the presence of cancer stem cells,^8^ representing a small population of cells within tumors. They have the capacity to self‐renew, proliferate, and differentiate 219 and contribute to the resistance to chemotherapeutic agents. They are the source of cells that give rise to distant metastases. Phenotypes comprising many cell surface markers have been identified for isolating cancer stem cells, including CD24, CD44, CD177, CD133, CD166, stage‐specific embryonic antigen‐1 (SSEA‐1), SSEA‐4, and ALDH1.^219^, ^220^ Accumulating evidence indicates that TCM is a promising strategy for the elimination of cancer stem cells.

As described by Zendehdel et al, curcumin can inhibit the viability of cancer stem cells using a variety of mechanisms, including prevention of inflammatory cytokine and transcription factor activation, inhibition of the activity of various protein kinases, suppression of cytokine signaling receptors and growth factors, modulation of the activity of enzymes participating in inflammation and tumorigenesis, and change in the expression of adhesion molecules, apoptotic proteins, and other targets.^222^ It has been found that curcumin destroys chemoresistant colon cancer stem cells by suppressing the expression of the cancer stem cell marker genes ALDH, CD44, CD133, and CD166.^38^ In fact, curcumin has been reported to significantly affect various types of cancer stem cell (Table 1).

A study of the effects of exposure to resveratrol in seven glioma stem cell lines isolated from glioblastoma multiforme patients also found that it inhibited the proliferation of glioma stem cells via modulation of the Wnt signaling pathway and c‐Myc expression.^57^ The effect of resveratrol in cervical cancer stem cells is to cause sensitization that induces apoptosis by inhibiting RAD51 expression.^58^ In breast cancer, resveratrol prevents cancer stem cell‐like characteristics by promoting the expression of a number of tumor‐suppressing miRNAs, including miR‐16, −141, −143, and −200c.^59^ Resveratrol has been demonstrated to have efficacy against colon cancer stem cells through elevation of p53 levels, the Bax/Bcl‐2 ratio, and cleavage of PARP.^60^ Furthermore, resveratrol sensitizes leukemia stem cell‐like KG‐1a cells to cytokine‐induced killer cell‐mediated cytolysis through an increase in cell surface expression of NKG2D ligands and activation of the TRAIL pathway.^61^ In summary, resveratrol is widely used in the treatment of cancer stem cells.

Treatment with berberine has been shown to reduce the activity of ALDH1, capability for self‐renewal and metastasis, and the chemosensitivity of oral squamous cell carcinoma stem cells in a dose‐dependent manner.^71^ Berberine‐filled liposomes have been reported to cross the cell membrane of cancer stem cells, suppressing ABC transporters and regulating apoptosis‐related signaling pathways.^223^ However, there are fewer reports of treatment of cancer stem cells with berberine than with curcumin or resveratrol.

### 6.2 TCM for prevention of EMT and metastasis

It has been reported that breast cancer stem cells originate from either normal mammary stem cells or mammary epithelial cells via an EMT process.^219^ EMT is a fundamental mechanism that occurs during embryonic development and tissue differentiation, referring to the progressive loss of epithelial cell characteristics and the acquisition of mesenchymal features and stem‐like properties. EMT is an early step leading to solid tumor progression and is implicated in tumor growth, invasion, metastasis, and resistance to therapy. It contributes to the conversion of tumors from low‐ to high‐grade malignancy.^224^, ^225^ EMT, cancer stem cells and the microenvironment are closely related. The tumor microenvironment can instruct carcinoma cells to undergo EMT to initiate local invasion and metastatic dissemination.^214^ It has been suggested that activation of an EMT program leads these cells to acquire cancer stem cell properties.^224^ Therefore, targeting the signals involved in EMT has been shown to halt tumor progression in various human cancers.

Curcumin can block β‐catenin nuclear translocation and restore the expression of E‐cadherin and so amplify E‐cadherin/β‐catenin complex formation and β‐catenin cytosolic retention, thus suppressing EMT and migration of breast cancer stem cells.^226^ In colorectal cancer, curcumin inhibits EMT progression by enhancing E‐cadherin expression, decreasing vimentin expression, and regulation of the NKD2‐Wnt‐CXCR4 signaling pathway.^227^ A large number of studies have been published that document the influence of treatment with curcumin on the EMT process and metastasis.

Resveratrol has also been shown to inhibit the migration and invasion of cancer stem cells by downregulation of the EMT process. In oral squamous cell carcinoma cells, resveratrol can suppress cell invasion and migration by decreasing the expression of EMT‐inducing transcription factors.^228^ During cancer chemotherapy, acquired Doxorubicin resistance severely impedes the therapeutic effect, invariably leading to poor prognosis. In gastric cancer, resveratrol can reverse Doxorubicin resistance by interfering with the EMT process via modulation of PTEN/Akt signaling.^229^ Resveratrol appears to be a potent inhibitor of EMT and metastasis in cancer.

The transforming growth factor‐β (TGF‐β) signaling pathway regulates proliferation, invasion, migration, and EMT in cancer cells. Berberine is able to reduce the expression of TGF‐β1, cell migration, and in vivo tumor growth in breast and lung cancer.^230^ In nasopharyngeal carcinomas, berberine inhibits TGF‐β‐induced tumor invasion and the EMT process by regulation of E‐cadherin expression and suppression of Sp1 expression.^162^ Additionally, berberine attenuates the expression of the cancer stemness marker CD133, upregulates the mesenchymal markers vimentin and fibronectin, and restores the epithelial marker E‐cadherin in neuroblastoma,^231^ indicating that berberine can possibly block the cross‐talk between cells undergoing EMT and cancer stem cells, thus inhibiting cancer development and recurrence.

## 7 CONCLUSIONS AND PERSPECTIVE

In recent decades, increasing numbers of patients have been attracted to use TCM as an adjuvant therapy option for various diseases. In particular, TCM‐based CHM has increasingly been shown to exhibit promising therapeutic effects as an adjunctive treatment following surgery, chemotherapy, radiotherapy, or other types of therapy for cancer patients worldwide. CHM is considered a gift of nature and these compounds derived from herbs have the advantage of availability, efficacy, and relatively low toxicity compared with chemotherapy. Evidence has confirmed that TCM in combination with chemotherapy or radiotherapy is capable of promoting the efficacy of and diminishing the limitations and drawbacks induced by chemotherapy and radiotherapy.^232^ A considerable quantity of research has been performed to elucidate the mechanisms underlying the beneficial effects of TCM in cancer treatment. As mentioned above, TCM can possibly regulate oncogenes and tumor suppressor genes, epigenetic modification, the microenvironment, and cancer stem cells. The objective of this review was to contribute to a clearer understanding of TCM as an adjuvant therapy for cancer. The number of studies on curcumin and resveratrol in cancer therapy are in much greater abundance than other CHM‐derived phytochemicals, so there is much to learn about those compounds. In order to apply the full potential of TCM in cancer therapy and broaden its application, the underlying mechanisms of many more CHM‐derived compounds must be further exploited.

## CONFLICT OF INTEREST

The authors declare that they have no conflict of interest.
